# Preference for Exercise vs. More Sedentary Reinforcers: Validation of an Animal Model of Tetrabenazine-Induced Anergia

**DOI:** 10.3389/fnbeh.2019.00289

**Published:** 2020-01-30

**Authors:** Carla Carratalá-Ros, Laura López-Cruz, Noemí SanMiguel, Patricia Ibáñez-Marín, Andrea Martínez-Verdú, John D. Salamone, Mercè Correa

**Affiliations:** ^1^Àrea de Psicobiologia, Campus de Riu Sec, Universitat Jaume I, Castelló de la Plana, Spain; ^2^Behavioral Neuroscience Division, University of Connecticut, Storrs, CT, United States

**Keywords:** dopamine, accumbens, voluntary exercise, reward, depression, binge eating, aversion

## Abstract

Physical activities can have intrinsic motivational or reinforcing properties. The choice to engage in voluntary physical activity is undertaken in relation to the selection of other alternatives, such as sedentary behaviors, drugs, or food intake. The mesolimbic dopamine (DA) system plays a critical role in behavioral activation or exertion of effort, and DA antagonism or depletion induces anergia in effort-based decision-making tasks. However, little is known about the neural mechanisms underlying the decision-making processes that establish preferences for sedentary vs. activity-based reinforcers. In the present work with male CD1 mice, we evaluated the effect of tetrabenazine (TBZ), a DA-depleting agent, on a three-choice T-maze task developed to assess preference between reinforcers with different behavioral activation requirements and sensory properties [i.e., a running wheel (RW) vs. sweet pellets or a neutral nonsocial odor]. We also studied the effects of TBZ on the forced swim test (FST), which measures climbing and swimming in a stressful setting, and on anxiety tests [dark-light (DL) box and elevated plus maze (EPM)]. In the three-choice task, TBZ reduced time running in the wheel but increased time spent consuming sucrose, thus indicating reduced activation but relatively intact sucrose reinforcement. The effect of TBZ was not mimicked by motivational manipulations that change the value of the reinforcers, such as making the RW aversive or harder to move, food-restricting the animals, inducing a binge-like eating pattern, or introducing social odors. In the FST, TBZ decreased time climbing (most active behavior) and increased immobility but did not affect anxiety in the DL or EPM. These results indicate that the three-choice T-maze task could be useful for assessing DA modulation of preferences for exercise based on activation and effort requirements, differentiating those effects from changes in preference produced by altering physical requirements, food restriction state, and stress during testing.

## Introduction

Motivated behavior is characterized by a high degree of behavioral activation, as demonstrated by the speed, vigor, or persistence seen in the instigation and maintenance of instrumental responding (Salamone and Correa, 2002, 2012; Robbins and Everitt, 2007; Mai et al., 2012; McGinty et al., 2013; Floresco, 2015). Animal research has demonstrated that this activational aspect of motivation is partly regulated by the mesolimbic dopamine (DA) system (Salamone et al., 2006, 2016, 2018). In particular, nucleus accumbens DA and related neural systems have been implicated in motivational dysfunctions such as anergia and fatigue seen in many neurological and psychological pathologies (Salamone and Correa, 2002, 2012; Stahl, 2002; Treadway et al., 2012).

Diverse tasks have been used in rodents for evaluating behavioral activation and effort-related decision making, including tasks that give animals the option of vigorously working (lever pressing or climbing a barrier) to obtain access to more highly valued reinforcers vs. approaching and consuming a less preferred reinforcer (Cousins et al., 1994; Salamone and Correa, 2002; Salamone et al., 2016; Mott et al., 2009; Mai et al., 2012; Pardo et al., 2012, 2015; Randall et al., 2012; Sommer et al., 2014; Yohn et al., 2015a, 2016b; Correa et al., 2018; SanMiguel et al., 2019). In these tasks, conditions that alter DA transmission, such as administration of DA antagonists or tetrabenazine (TBZ), can alter behavioral activation and reduce selection of high-effort choices in rats (Nunes et al., 2013; Randall et al., 2014; Hosking et al., 2015; Pardo et al., 2015; Yohn et al., 2015a, 2016a,b; Contreras-Mora et al., 2018; Rotolo et al., 2019). TBZ acts by inhibiting the vesicular monoamine transporter-type 2 (VMAT-2), which leads to a blockade of vesicular storage and a depletion of monoamines, with its greatest effects at low doses being on striatal DA in rats and mice (Pettibone et al., 1984; Nunes et al., 2013; López-Cruz et al., 2018). In humans, TBZ is used to treat Huntington’s disease, but major side effects include depressive symptoms, including fatigue and depression (Frank, 2009; Guay, 2010; Chen et al., 2012). In fact, TBZ administered chronically in a mouse model of Huntington’s disease (Wang et al., 2010) improved motor deficits but increased depression-like measures in the forced swim test (FST).

The FST is the classical rodent task for assessing the antidepressant properties of many substances. This test is based on the observation that rodents exposed to a stressful non-escapable situation such a deep tank full of water initially try to escape in a vigorous way, but eventually, they will cease these vigorous attempts and will instead passively float in the chamber (Porsolt et al., 1977). Thus, immobility is the classical parameter that is typically measured, and antidepressant drugs have repeatedly been shown to reduce immobility time (Porsolt et al., 1977; Armario et al., 1988; Costa et al., 2013). However, escape-related mobility such as climbing or struggling is an active behavior that also is modified by antidepressant drugs (Armario et al., 1988; Lucki, 1997). These active behaviors are more likely to be affected by DA manipulations than the traditional immobility measure (Gil and Armario, 1998; Costa et al., 2013). Thus, drugs that affect dopaminergic transmission such as haloperidol have been shown to decrease climbing or struggling behavior in rats (Gil and Armario, 1998). However, TBZ has not been assessed in relation to active behaviors such as climbing or struggling in the FST.

Recently, a T-maze choice task has been developed that assesses the impact of drugs on behavioral activation and effort-related choice (Correa et al., 2016; López-Cruz et al., 2018). This task allows the animal to freely choose between running on a wheel, consuming sucrose pellets, or sniffing a neutral nonsocial odor. Although foods high in sucrose are generally highly preferred, inducing strong hedonic reactivity in rodents (Berridge, 2000; Levine et al., 2003), voluntary wheel running occurs in mice of all strains, sexes, and ages (Walker and Mason, 2018). Previous studies in our laboratory have demonstrated that adult male CD1 mice strongly preferred the running wheel (RW) in the T-maze choice task and that DA antagonism or DA depletion partially shifted choice behavior, reducing time running but increasing time eating sucrose (Correa et al., 2016; López-Cruz et al., 2018). This T-maze task does not involve the use of food restriction, as is the case with other effort-based choice tasks like the T-maze barrier-climbing task for food reinforcement (Pardo et al., 2012; Correa et al., 2018), and does not involve stressful conditions, such as the FST. Moreover, since mice have a high preference for engaging in physical activities (Routtenberg, 1968; Correa et al., 2016; López-Cruz et al., 2018), the present task offers a good test for the evaluation of the brain mechanisms involved in the decision-making processes that establish spontaneous preference for active and vigorous behaviors.

In the present work, we explored the impact of a broad range of TBZ doses that have been demonstrated to deplete accumbens DA in mice (López-Cruz et al., 2018), comparing its impact on different measures of behavioral activation induced by stressful conditions (FST) or by spontaneous preference for physical activity in the T-maze choice task. In addition, we explored if the ability of TBZ to reduce RW preference and increase palatable food preference can be mimicked by manipulating reinforcement value of the various conditions. Thus, we did a series of behavioral manipulations that attempted to increase the value of the sweet pellets by food-restricting the animals prior to the T-maze tests or, in another group of animals, by inducing a binge-like eating pattern after randomly presenting sweet pellets during short sessions several days a week previously to the T-maze sessions. We also increased the potential value of the odor stimulus by using conspecific same- or different-sex odors. Finally, we reduced RW value by increasing the resistance of the wheel or introducing an anxiogenic light over the RW. The effect of TBZ on anxiety paradigms [dark-light (DL) box and elevated plus maze (EPM)] was also assessed in order to identify anxiolytic-like actions that could be affecting the results in the T-maze and the FST.

## Materials and Methods

### Animals

CD1 adult male mice (*N* = 166) purchased from Janvier, France S.A., were 8–10 weeks old (30–40 g) at the beginning of the study. Mice were housed in groups of three or four per cage, with standard laboratory rodent chow and tap water available *ad libitum*. The colony was kept at a temperature of 22 ± 2°C with lights on from 08:00 to 20:00 h. All procedures were covered by a protocol approved by the Institutional Animal Care and Use Committee of the Universitat Jaume I. All experimental procedures complied with directive 2010/63/EU of the European Parliament and of the Council and with the “Guidelines for the Care and Use of Mammals in Neuroscience and Behavioral Research,” National Research Council 2003, USA. All efforts were made to minimize animal suffering and to reduce the number of animals used.

### Pharmacological Agents

TBZ (Cymit Quimica SL, Spain) was dissolved in a vehicle solution of 0.9% saline (80%) plus dimethyl sulfoxide (DMSO 20%, final pH 5.5) and administered 120 min before testing. Time elapsed after injection and range of doses were selected based on previous work (Correa et al., 2018; López-Cruz et al., 2018) and pilot studies demonstrating that in mice, these are an optimal dose and time lead to deplete DA. DMSO (20% v/v) was used as the control group. TBZ and DMSO were administered intraperitoneally (IP).

### Testing Procedures

All behavioral procedures started 2 h after the light-period onset. The behavioral test room was illuminated with a soft light, and external noise was attenuated.

#### T-Maze RW–Sucrose–Odor Choice Task

**Figure 1:**
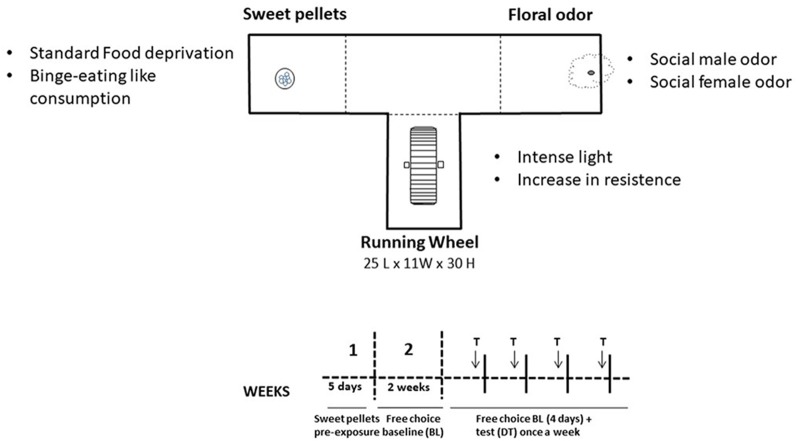
Schematic representation of the three-choice T-maze task settings and timeline for the different experimental phases for the tetrabenazine (TBZ) experiment.

The T-maze apparatus consisted of a central area that leads to three arms (see Figure 1). In one of them, sucrose pellets (TestDiet™, 50% sucrose, 45 mg each) were available; in another arm, there was an RW; and in the third arm, there was a hole with a cotton ball socked with a fruit odor (based on López-Cruz et al., 2018). This concentration of sucrose in the pellets was selected after piloting different concentrations (i.e., 100% sucrose pellets generate avoidance), and the nonsocial odor used for the present studies (strawberry) was the one that generated more exploration among nonsocial odors in previous studies (López-Cruz et al., 2017). In general, mice were allowed to freely explore and interact with the stimulus during 15-min sessions, once a day, 5 days a week. In training phase 1, to avoid neophobia to the sweet-tasting pellets, animals were enclosed in that arm with the food during five sessions (no exploration of the other arms was allowed during this phase). In training phase 2, during 2 weeks, animals had free access to the three stimuli until a stable baseline (BL) was obtained. The test phase lasted four more weeks. For each week, there were four BL drug-free sessions plus a testing session in which animals received TBZ or were tested under different behavioral manipulations. The day before the test for the behavioral manipulation was considered as the BL for those experiments. Sessions were videotaped, and a trained observer manually registered several parameters. Time interacting with the stimulus was selected as the main dependent measure because it allowed for the evaluation of the three stimuli with the same units. Time allocation is a useful measure of preference, relative reinforcement value, and response choice (Baum and Rachlin, 1969). Entries into the arms and time spent in the arms of the T-maze were also simultaneously recorded. All these measures were taken based on previous studies (Correa et al., 2016; López-Cruz et al., 2018).

#### Forced Swim Test

This paradigm is considered to be a model of behavioral despair and is used as a test for assessing depressive-like states (Porsolt et al., 1977). Naïve mice were placed in a transparent cylindrical glass tank (26 cm high and 18 cm in diameter) filled with water (14 cm) and maintained at a temperature of 25°C. Water was changed between animals. During the 6-min test, mice were videotaped from the side, and struggling/climbing, immobility, and swimming were later measured by an observer unaware of the experimental condition. Immobility was defined as a period when the animal remained motionless, making only minor movements to balance the body and keep the head above the water. In addition, we also assessed escape-related mobility behavior such as climbing or struggling (Armario et al., 1988). Climbing is defined as any energetic and vertical movement of all four limbs against the wall of the tank. Swimming was recorded when animals carried out horizontal movements with their forepaws, leading to the displacement of the body throughout the swim chamber (Armario et al., 1988). After the test, mice were dried with a soft towel, put back in a box with absorbing paper under a warming light, and monitored for 10 min.

#### DL Box

The DL box test is based on the conflict between the tendency to explore a novel environment and the avoidance of a brightly lighted open area (Blumstein and Crawley, 1983). The DL box apparatus consisted of a polypropylene chamber divided into two compartments by a partition containing a small opening (5 cm H × 5 cm W). The light compartment (25 cm W × 25 cm H × 25 cm L) was open, painted in white, and illuminated with an intense light (335 lx), while the dark compartment (25 cm W × 25 cm H × 18 cm L) was painted in black and had a removable ceiling to close it (Kulesskaya and Voikar, 2014). To start the test session, mice were individually placed in the dark chamber facing one corner. Test sessions were videotaped. Total number of crosses between compartments as an index of locomotion and total time spent in the lit chamber as an index of anxiogenesis were recorded for 5 min (the procedure is based on López-Cruz et al., 2014).

#### Elevated Plus Maze

After being in the DL box for 5 min, animals were placed in the EPM for five more minutes. The EPM consists of two open and two enclosed arms (65 cm L × 5 cm W) arranged in a plus configuration with an intersection in a central platform. It was made of black polypropylene and is elevated 50 cm above the floor. The open arms had a 1-cm border around their perimeter, and the closed arms had a 20-cm translucent wall. This anxiety paradigm measures the avoidance that rodents show to elevated open spaces (Lister, 1987; Walf and Frye, 2007). Under normal conditions, mice spend more time and make more entries into the closed arms of the maze. Animals were placed in the central platform with their head pointing at one enclosed arm, and they were assessed for 5 min. Sessions were videotaped, and a trained observer registered the total time spent in the open arms and total entries in the four arms as an index of locomotion. An entry into an arm was recorded when the animal crossed, with all four legs, the line that connected that arm with the central platform (this procedure is based on López-Cruz et al., 2014).

### Experiments

Table 1 summarizes the experiments conducted, the experimental design, and number of mice used in each experiment.

**Table 1:** Experimental design and number of mice used in each experiment (FST, forced swim test; DL box, dark-light box; EPM, elevated plus maze).

Pharmacological or behavioral manipulation	Experimental design	Control condition
**TBZ effect**
T-maze	*N* = 13. Within groups	Vehicle day
FST	*N* = 47. Between groups	Vehicle group
DL, EPM	*N* = 41. Between groups	Vehicle group
**Change in relative value of sweet pellets**		
Standard food restriction	*N* = 8. Within groups	BL with no food restriction
Binge eating	*N* = 25. Between groups	Group exposed to standard food
**Change in the relative value of the odor**		
Social odors	*N* = 10. Within groups	BL with nonsocial odor
**Change in the relative value of the RW**		
Increase in RW resistance	*N* = 8. Within groups	BL with no extra weight
Light over the RW	*N* = 14. Within groups	BL with no light over RW

#### Experiment 1

##### Effect of TBZ on Preference for Active Reinforcers as Measured in the Three-Choice-T-Maze Task

After reaching a stable BL level of performance in the T-maze, animals (*n* = 13) received the vehicle or the DA-depleting agent TBZ (vehicle, 4.0, 6.0, and 8.0 mg/kg) 120 min before the test began. Animals received one dose of the drug every week in a randomly varied order. In previous work from our laboratory using rats and mice, we have observed that TBZ does not produce sensitization or tolerance when administered once a week; animals recover their behavioral BL performance after a few hours of the acute intraperitoneal injection. The T-maze paradigm requires a BL performance of 2 weeks before tests start, and that performance is maintained across weeks, thus allowing a repeated-measures design.

#### Experiment 2: Manipulations That Change the Relative Value of the Sweet Pellets

##### Experiment 2.1: Effect of Standard Food Restriction in the Home Cage

Animals (*n* = 8) were trained, and after reaching stable levels, they were food-restricted to 4.0 mg of standard chow the night before the test, in order to increase their appetite for the sucrose pellets. This amount of standard food was selected based on previous studies using food restriction (Pardo et al., 2012; Yang et al., 2020). BL with *ad libitum* food was used as the control condition.

##### Experiment 2.2: Binge-Eating-Like Consumption of Sweet Pellets

Before the T-maze testing took place, during 4 weeks, at intermittent random days (3 days a week in average), non-food-restricted mice (*n* = 12) were placed individually in standard home cages with free *ad libitum* access to sweet pellets (50% sucrose) and water for 1 h during the light cycle (based on Murphy et al., 2018). The control group (*n* = 13) was also placed in the same type of cages for an hour with standard food and water. After this 4 weeks, mice were trained in the T-maze.

##### Experiment 2.3: Effect of TBZ and of Increasing Food Value on the Amount of Sweet Pellets Consumed in the T-maze

The effect of the highest dose of TBZ (8.0 mg/kg) vs. The vehicle from experiment 1 was used in order to make comparisons with the effects of food restriction and binge-like eating exposure on milligrams of sucrose pellets consumed on the test day. The effects of food restriction and binge-like eating exposure were compared to their respective control groups. Thus, all these data were obtained from experiments 3, 2.2, and 2.3.

#### Experiment 3: Manipulations That Change the Relative Value of the Odor: Effect of Social Odors

Animals (*n* = 10) were trained as in previous experiments, and on the test day, a social odor (bedding from male or from female conspecifics) replaced the strawberry odor during two consecutive days: in the first session, male conspecific odor was used, and the following day, female mice odor replaced the male odor. The estrous cycle of the females was not controlled. BL was taken with the nonsocial odor and was used as the control condition.

#### Experiment 4: Manipulations That Change the Relative Value of the RW

##### Experiment 4.1: Effect of Increasing RW Resistance

Mice (*n* = 8) were trained as in previous experiments. The test was performed during two consecutive days: in the first day, weights were attached to the wheel so that the resistance increased 75%, and for the second day, additional weights increased resistance to 95%. Before increasing the resistance of the wheel, the behavior of animals in the T-maze was established as BL and used as the control condition.

##### Experiment 4.2: Effect of an Intense Light Over the RW

Mice (*n* = 14) were trained normally in the T-maze, and for the test day a bright light (like the one used in the DL box) was placed over the RW in order to add an aversive component. BL with standard test lighting conditions was used as the control condition.

#### Experiment 5: Effect of TBZ on Depressive-Like Behaviors Assessed in the FST

Naïve animals (*n* = 47) received one dose of TBZ or vehicle (vehicle, 4.0, 6.0, or 8.0 mg/kg) and, 120 min after the injection, were placed in the FST. Mice were exposed only once to the FST since behavioral habituation develops in one session.

#### Experiment 6: Effect of TBZ on Anxiety Parameters as Measured in the DL and EPM Paradigms

Mice (*n* = 41) received one dose of TBZ (0, 4, 6, or 8 mg/kg) 120 min before the test began. Animals were first placed in the DL box for 5 min, and immediately after this test, they were placed in the EPM for five more minutes. Mice were exposed only once to both paradigms, since behavioral habituation develops in one session.

### Statistical Analyses

Normally distributed and homogenous data (according to the Kolmogorov–Smirnov test) for the FST, DL, and EPM experiments employed a between-groups design, and data were analyzed by one-way repeated-measures ANOVA. Data for the key dependent variable in the T-maze experiments, time with the three reinforcers, were analyzed by a MANOVA in previous work (Correa et al., 2016). Because the three-way interaction was significant, univariate ANOVAs were conducted for each reinforcer in all the dependent variables. Since, in the present work, BLs are consistent among the three stimuli and they are also consistent across experiments, we performed univariate ANOVAs. Thus, normally distributed data in the T-maze experiments followed a within groups-design with the exception of the binge-eating experiment, which used two different groups of animals. Thus, when more than two experimental conditions were used such as in the TBZ experiment, the experiment with increasing RW resistance, and the social odors experiments, data were analyzed by repeated-measures ANOVA. When the overall ANOVA was significant, non-orthogonal planned comparisons using the overall error term were used to compare each treatment with the vehicle control group (Keppel, 1991). For these comparisons, the α level was kept at 0.05 because the number of comparisons was restricted to the number of treatments minus 1. The effects of intense light over the RW and food restriction were evaluated by Student’s *t*-test for dependent samples. Binge-like eating of sweet food was analyzed by Student’s *t*-test for independent samples. All data were expressed as mean ± SEM, and significance was set at *p* < 0.05. Statistica 7 software was used.

## Results

### Experiment 1: Effect of TBZ on Preference for Active Reinforcers as Measured in the Three-Choice T-maze Task

**Figure 2:**
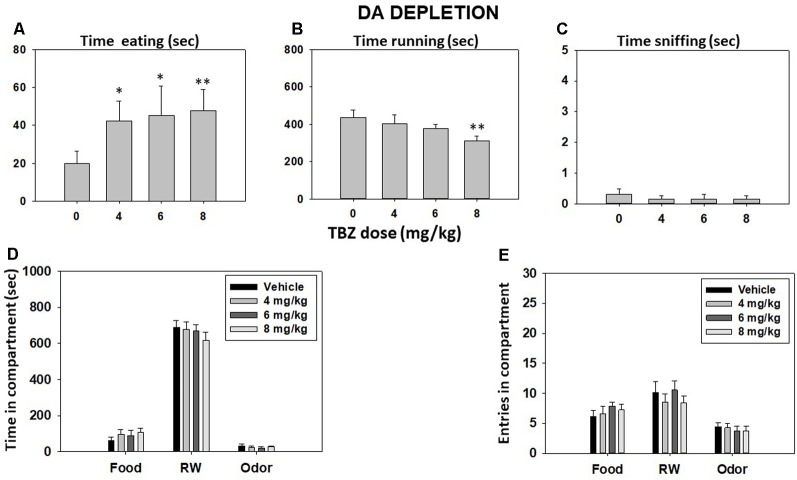
Effect of TBZ (vehicle and 4, 6, and 8 mg/kg) on time eating **(A)**, time running **(B)**, time sniffing **(C)**, time spent in each compartment **(D)**, and entries into compartments **(E)** in the T-maze task assessed during 15 min. Bars represent mean ± SEM of accumulated seconds or number of entries. *p < 0.05, **p < 0.01 significantly different from the vehicle.

Repeated-measures ANOVA showed that the total time spent sniffing the neutral odor was not significant (*F*_(3,36)_ = 0.29, *p* = 0.83). However, TBZ produced a significant effect on time spent eating (*F*_(3,36)_ = 3.46, *p* < 0.05) and time spent running in the RW (*F*_(3,36)_ = 2.99, *p* < 0.05; Figures 2A–C). Planned comparisons revealed that all doses of TBZ produced a significant increase in time consuming sucrose pellets in comparison with the vehicle group (*p* < 0.05 for 4.0 and 6.0 mg/kg and *p* < 0.01 for 8.0 mg/kg). Planned comparisons also showed that mice treated with 8.0 mg/kg TBZ spent significantly less time running in the RW compared with the vehicle group (*p* < 0.01). Repeated-measures ANOVA showed that total time spent in the RW compartment (*F*_(3,36)_ = 0.98, *p* = 0.40), the food compartment (*F*_(3,36)_ = 1.11, *p* = 0.35), and the neutral odor compartment (*F*_(3,36)_ = 0.89, *p* = 0.45) were not significant (Figure 2D). Finally, TBZ did not produce significant differences in the number of entries into the RW compartment (*F*_(3,36)_ = 0.98, *p* = 0.40), food compartment (*F*_(3,36)_ = 0.70, *p* = 0.55), and neutral odor compartment (*F*_(3,36)_ = 0.46, *p* = 0.71; data shown in Figure 2E).

### Experiment 2: Manipulations That Change the Relative Value of the Sweet Pellets

#### Experiment 2.1: Effect of Standard Food Restriction in the Home Cage

**Figure 3:**
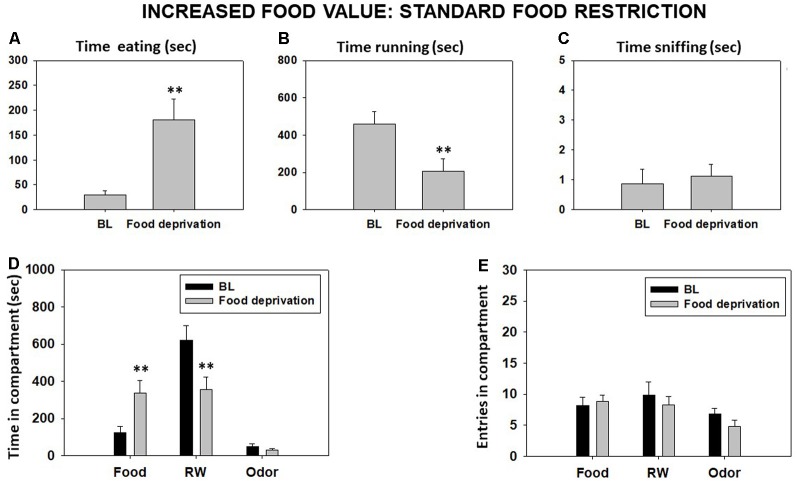
Effect of home food restriction in mice behavior in the T-maze task. Bars represent mean (±SEM) of time (seconds) spent eating **(A)**, running **(B)**, and sniffing the neutral odor **(C)** and time in different compartments **(D)** and entries **(E)** during a 15-min session. Bars represent mean ± SEM of accumulated seconds or number of entries. **p < 0.01 significantly different from baseline (BL).

Food restriction significantly decreased time in RW and significantly increased time eating compared with the BL condition as shown by Student’s *t*-test for dependent samples (*t* = 4.95, *p* < 0.01, and *t* = −4.24, *p* < 0.01, respectively). However, there was no significant difference between conditions on time spent sniffing the neutral odor (*t* = −0.42, *p* = 0.68; Figures 3A–C). Student’s *t*-tests for time in each compartment showed significant differences between both conditions in time spent in the food compartment (*t* = −3.10, *p* < 0.01) and RW compartment (*t* = 3.22, *p* < 0.01), but no significant effect on time in the odor compartment (*t* = 1.50, *p* = 0.17; Figure 3D). Finally, there were no significant differences in total entries to the food compartment (*t* = −0.84, *p* = 0.42), to the RW compartment (*t* = 0.30, *p* = 0.76), and to the odor compartment (*t* = 2.19, *p* = 0.06; Figure 3E).

#### Experiment 2.2: Binge-Like Consumption of Sweet Pellets

**Figure 4:**
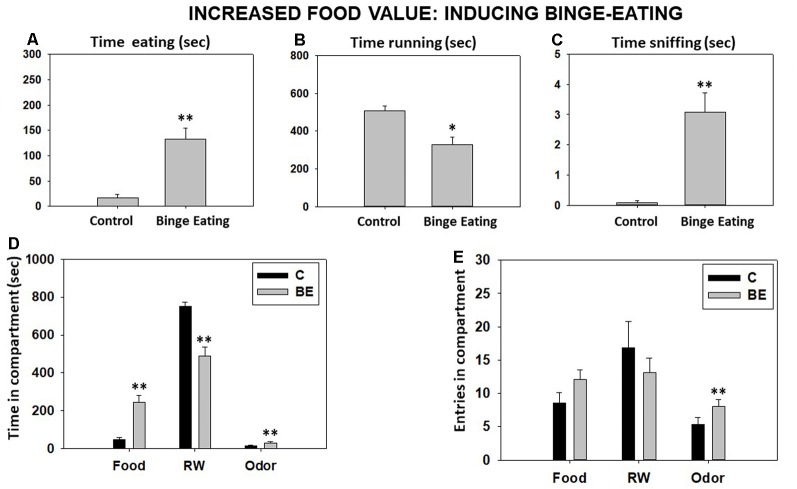
Effect of binge eating pattern for sucrose pellets on time eating **(A)**, time running **(B)**, time sniffing **(C)**, time spent in each compartment **(D)**, and compartment entries **(E)** in the T-maze task assessed during 15 min. Bars represent mean ± SEM of accumulated seconds or number of entries. *p < 0.05, **p < 0.01 significantly different from the control group.

Student’s *t*-test for independent samples showed a significant increase in time eating sucrose pellets (*t* = −5.60, *p* < 0.01) and time sniffing the neutral odor (*t* = −4.68, *p* < 0.01) but a significant decrease in time running in the RW (*t* = 3.92, *p* < 0.05) compared with the BL condition (Figures 4A–C). Moreover, Student’s *t*-test for time in each compartment showed significant differences between both conditions for time spent in the food compartment (*t* = 5.60, *p* < 0.01), the RW compartment (*t* = 5.99, *p* < 0.01), and the odor compartment (*t* = 4.68, *p* < 0.01; Figure 4D). Finally, there were no significant differences in total entries to the food compartment (*t* = −1.88, *p* = 0.08) and the RW compartment (*t* = 1.06, *p* = 0.30), but there was a significant increase in total entries to the odor compartment (*t* = −2.75, *p* < 0.01; Figure 4E).

#### Experiment 2.3: Effect of TBZ and Changes in Food Value on the Amount of Sweet Pellets Consumed in the T-maze

Student’s *t*-test for dependent samples did not show significant differences between the vehicle condition and the 8.0-mg/kg TBZ condition (*t* = −0.75, *p* = 0.46). However, Student’s *t*-test for dependent samples showed that there were significant differences among conditions in the experiment in which animals were food deprived (*t* = 3.05, *p* < 0.01). Similarly, Student’s *t*-test for independent samples showed significant differences between the control group and the binge-like eating group (*t* = −3.84, *p* < 0.01). Data are shown in Table 2.

**Table 2:** Effect of tetrabenazine (TBZ) and behavioral manipulations that increased food value on pellets consumed in the T-maze.

	Pellet intake (mg)	
	Control	Experimental condition
TBZ (vehicle and 8.0 mg/kg)	169.6 ± 48.1	228.4 ± 50.5
Food restriction	157.7 ± 32.9	736.1 ± 235.8**
Binge eating	301.1 ± 61.4	684.6 ± 82.5**

Mean (±SEM) of milligrams consumed. **p < 0.01 significantly different from their corresponding control condition.

### Experiment 3: Manipulations That Change the Relative Value of the Odor: Effect of Social Odors

**Figure 5:**
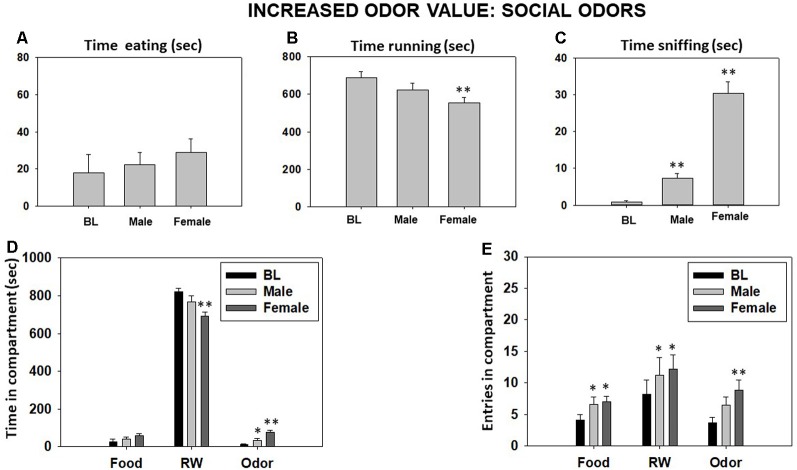
Effect of changing from fruit odor to social odors in the odor compartment of the T-maze task on time eating **(A)**, time running **(B)**, time sniffing **(C)**, time spent in each compartment **(D)**, and entries into compartments **(E)** in the T-maze task assessed during 15 min. Bars represent mean ± SEM of accumulated seconds or number of entries. *p < 0.05, **p < 0.01 significantly different from BL.

Repeated-measures ANOVA showed an overall effect of odor type on time sniffing (*F*_(2,18)_ = 85.98, *p* < 0.01). Planned comparisons revealed a significant increase in time sniffing the same-sex conspecific and female odors (both *p* < 0.01; Figure 5C). The ANOVA also yielded an overall effect on time running in the RW (*F*_(2,18)_ = 7.34, *p* < 0.01), and the planned comparisons showed a significant difference between BL and the female odor condition (*p* < 0.01; Figure 5B). However, there was not an effect of type of odor on time spent eating (*F*_(2,18)_ = 0.61, *p* = 0.55; Figure 5A). For the time spent in the various compartments, the repeated-measures ANOVA showed an overall effect on time in the odor compartment (*F*_(2,18)_ = 23.45, *p* < 0.01) and time in the RW compartment (*F*_(2,18)_ = 8.99, *p* < 0.01). Planned comparisons demonstrated a significant increase in time spent in the odor compartment when the odor was male or female (*p* < 0.05 and *p* < 0.01, respectively), and there was a significant decrease in time spent in the RW compartment only when the odor was female (*p* < 0.01). There was not an effect of odor type on time spent in the food compartment (*F*_(2,18)_ = 2.08, *p* = 0.15; Figure 5D). Finally, repeated-measures ANOVA showed an overall effect of social odors on entries to the RW compartment (*F*_(2,18)_ = 4.31, *p* < 0.05), entries to the food compartment (*F*_(2,18)_ = 4.50, *p* < 0.05), and entries to the odor compartment (*F*_(2,18)_ = 6.35, *p* < 0.01). Planned comparisons showed an increase in entries to the food compartment when the male or the female odors were used as compared with the BL (*p* < 0.05), and the same was observed for the compartment where the odor was present (*p* < 0.05 for the male odor and *p* < 0.01 for the female odor both compared to BL). However, the total entries to the RW compartment increased only when the female odor was present in comparison with the neutral odor condition (*p* < 0.05; Figure 5E).

### Experiment 4: Manipulations That Changed the Relative Value of the RW

#### Experiment 4.1: Effect of Increasing RW Resistance

**Figure 6:**
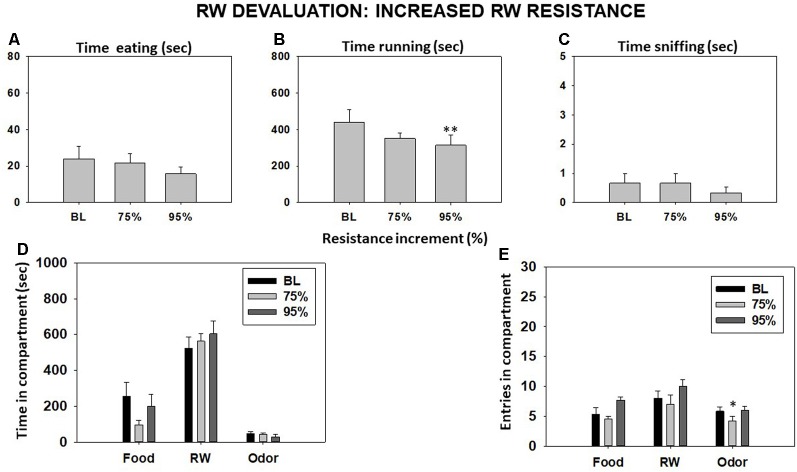
Effect of increasing running wheel (RW) resistance on mice behavior in the T-maze task. Bars represent mean (±SEM) of time (s) eating **(A)**, running **(B)**, and sniffing the neutral odor **(C)**; time spent in each compartment **(D)**; and compartment entries **(E)** during a 15-min session. Bars represent mean ± SEM of accumulated seconds or number of entries. *p < 0.05, **p < 0.01 significantly different from BL.

Repeated-measures ANOVA did not show a significant effect of increasing RW resistance on time eating (*F*_(2,10)_ = 1.03, *p* = 0.38) or time sniffing the neutral odor (*F*_(2,10)_ = 0.40, *p* = 0.68). However, RW resistance produced a significant effect on time running in the RW (*F*_(2,10)_ = 5.12, *p* < 0.05). Planned comparison showed a decrease in time running in the RW when the resistance was the highest (95%) in comparison with BL condition (*p* < 0.01; Figures 6A–C). Increasing the RW resistance did not yield a significant effect on time spent in the food compartment (*F*_(2,10)_ = 3.71, *p* = 0.06), time spent in the RW compartment (*F*_(2,10)_ = 2.12, *p* = 0.17), or time spent in the odor compartment (*F*_(2,10)_ = 2.64, *p* = 0.11; Figure 6D). Although repeated-measures ANOVA did not show an overall effect of increasing RW resistance on total entries to the RW compartment (*F*_(2,10)_ = 0.96, *p* = 0.40), it did yield a significant effect on entries to the food compartment (*F*_(2,10)_ = 4.89, *p* < 0.05) and to the odor compartment (*F*_(2,10)_ = 10.88, *p* < 0.01). Planned comparisons showed a decrease in total entries after applying 75% more resistance to the RW in comparison with the BL condition in the odor compartment (Figure 6E).

#### Experiment 4.2: Effect of an Intense Light Over the RW

**Figure 7:**
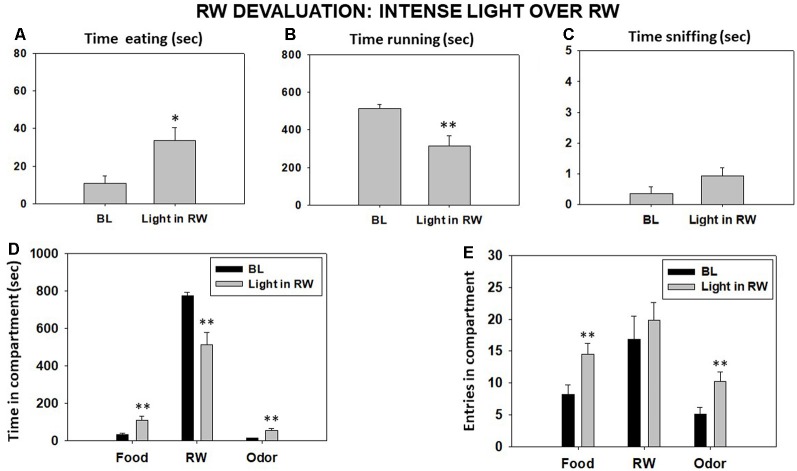
Effect of placing an intense light over the RW on time eating **(A)**, time running **(B)**, time sniffing **(C)**, time spent in each compartment **(D)**, and entries into compartments **(E)** in the T-maze task assessed during 15 min. Bars represent mean ± SEM of accumulated seconds or number of entries. *p < 0.05, **p < 0.01 significantly different from BL.

The bright-light condition significantly decreased the time that animals spent running in the RW (*t* = 2.98, *p* < 0.01) and increased the time they spent eating the sucrose pellets compared with the BL condition (*t* = −2.38, *p* < 0.05) as shown by Student’s *t*-test for dependent samples. However, the increase in sniffing time did not reach statistical significance (*t* = −1.37, *p* = 0.19; Figures 7A–C). Student’s *t*-test for time in these compartments showed a significant decrease in time spent in the RW compartment (*t* = 3.44, *p* < 0.01) and a significant increase in time spent in the food (*t* = −2.89, *p* < 0.01) and odor (*t* = −3.60, *p* < 0.01) compartments (Figure 7D). Finally, the *t*-tests for dependent samples showed an increase on entries to the food compartment (*t* = −4.03, *p* < 0.01) and to the odor compartment (*t* = −3.14, *p* < 0.01), although there were no significant differences in entries to the RW compartment compared with the BL condition (*t* = −0.83, *p* = 0.41; Figure 7E).

### Experiment 5: Effect of TBZ on Depressive-Like Behaviors Assessed in the FST

**Figure 8:**
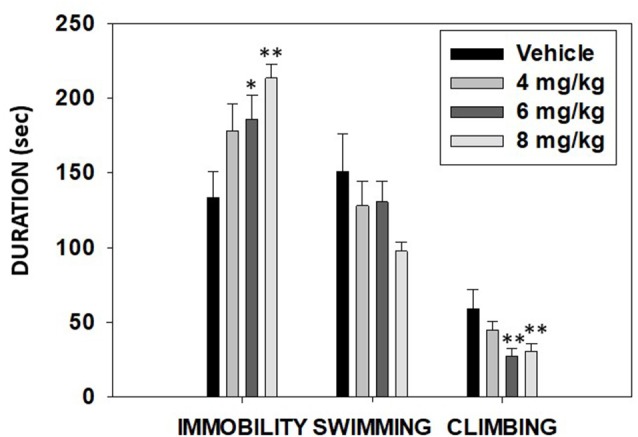
Effect of TBZ (vehicle and 4, 6, or 8 mg/kg) on duration of immobility, swimming, and climbing behavior in the forced swim test (FST) assessed during 6 min. Bars represent mean ± SEM of accumulated seconds. *p < 0.05, **p < 0.01 significantly different from the vehicle.

The ANOVA for time spent swimming did not show significance (*F*_(3,43)_ = 1.90, *p* = 0.14). However, TBZ produced a significant effect on immobility (*F*_(3,43)_ = 4.02, *p* < 0.01) and on climbing (*F*_(3,43)_ = 3.86, *p* < 0.01). Planned comparisons revealed that the groups that received the two highest doses of TBZ (6.0 and 8.0 mg/kg) displayed significantly more time immobile (*p* < 0.05 and *p* < 0.01, respectively) and less climbing (*p* < 0.01) in comparison with the vehicle group (Figure 8).

### Experiment 6: Effect of TBZ on Anxiety Parameters as Measured in the DL and EPM Paradigms

The ANOVAs did not show any significant effects on time spent in the illuminated area of the DL box (*F*_(3,37)_ = 2.22, *p* = 0.10) or time spent in the open arms (*F*_(3,38)_ = 0.68, *p* = 0.56) of the EPM, both of which are indices of anxiety. However, TBZ did have a significant effect on the total number of crosses between compartments in the DL box (*F*_(3,37)_ = 5.69, *p* < 0.01) and total number of crosses to all arms in the EPM (*F*_(3,38)_ = 6.38, *p* < 0.01). Planned comparisons revealed that the groups that received 6.0- and 8.0-mg/kg TBZ displayed significantly fewer crosses than did the vehicle group (*p* < 0.01) in the DL box, but only the group that received the highest dose of TBZ (8.0 mg/kg) displayed fewer crosses (*p* < 0.01) than the vehicle group in the EPM. The total number of crosses assessed in the DL box and EPM can be considered as an index of locomotion. Data are shown in Table 3.

**Table 3:** Effect of tetrabenazine (TBZ) on mice behavior in the dark-light (DL) box and elevated plus maze (EPM).

	Vehicle	TBZ (mg/kg)		
		4	6	8
**DL**				
Time in light compartment (s)	125.6 ± 6.1	116.8 ± 8.9	84.5 ± 13.5	117.1 ± 11.4
Crosses between compartments	23.6 ± 1.6	20.2 ± 1.8	17.2 ± 4.1**	12.6 ± 1.4**
**EPM**				
Time in open arms (s)	79.2 ± 13.7	75.2 ± 13.9	82.2 ± 8.5	95.9 ± 12.6
Crosses between arms	19.1 ± 1.0	15.2 ± 2.1	18.9 ± 1.5	11.2 ± 1.4**

Every test lasted 5 min. Mean (±SEM) seconds or frequency. **p < 0.01 significantly different from the vehicle.

## Discussion

The present group of studies evaluated the impact of the VMAT-2 inhibitor TBZ on behavioral activation as assessed in a three-choice T-maze task that evaluates preference for vigorous physical activity vs. other sources of reinforcement that could be obtained with little effort. These experiments also studied how TBZ can affect behavioral activation induced by stressful conditions such as the non-escapable FST.

In the three-choice T-maze task, mice freely distribute their time performing an effortful activity (running in a RW) or doing more sedentary activities (eating sucrose pellets or sniffing a fruit odor). Under basal conditions, mice spent a large part of the time running, some time consuming sucrose, and very little time sniffing a nonsocial odor. Consistent with this finding, previous studies have shown that running has a high motivational value, because animals work to unlock a RW (Collier et al., 1990; Belke et al., 2005; Belke and Pierce, 2014). Also, the RW can be used as a motivational stimulus for inducing conditioned place preference (Lett et al., 2000; Trost and Hauber, 2014; Basso and Morrell, 2015). In our study, TBZ decreased time spent running but increased time eating, with no change in time interacting with the neutral odor. These results are consistent with previous studies in our laboratory using lower doses of TBZ (López-Cruz et al., 2018) and also using haloperidol (D2 receptor antagonist), in a simpler version of the T-maze (Correa et al., 2016). In addition, in the present study, we further analyzed other behavioral variables such as time spent in the different compartments or total number of entries into each compartment. TBZ produced no significant change in these measures of place preference and exploration, indicating that mice did not avoid being in close proximity to the RW and showed normal motor exploration of the T-maze. A further statistical comparison of vehicle vs. the highest dose of TBZ with Student’s *t*-test for related samples did not show a significant effect on these two variables either. Thus, by depleting DA (López-Cruz et al., 2018), these doses of TBZ seem to be producing anergia or reduced behavioral activation rather than affecting the primary reinforcing effects of sucrose, since time spent eating sucrose actually increased.

However, in order to test potential alternative explanations, we manipulated the reinforcing value of these three reinforcers. Thus, we addressed potential factors that could be increasing food value. In one of the experiments, animals were exposed to the T-maze after having gone through home food restriction, in order to increase homeostatic value for food and food seeking. In this experiment, time spent eating was significantly increased almost 10 times, and time running decreased by half the BL values. Thus, this manipulation profoundly changed BL preferences, and it was the only manipulation that made RW and food equally preferred (mice spent around 200 s interacting with each one of them). In a different experiment, animals exposed daily to an unlimited amount of sweet food for a limited time demonstrated a binge eating pattern increasing about 10 times their time eating in the T-maze compared to control animals, almost to the level of animals in the previous experiment restricted of standard food. In addition, binge eating animals not only showed reduced time spent running but also showed increased time sniffing the strawberry odor. This pattern of results was parallel to results for the variable time in the compartment and for entries in the compartments, although only entries in the odor compartment increased significantly. These results indicate that a binge-like eating pattern produces a shift in relative preferences towards sedentary stimuli (odor and food). However, when we analyzed the amount of pellets consumed after DA depletion and after food manipulations (Table 2), we observed that animals significantly consumed more grams of sucrose pellets only after food restriction and after establishing a binge-like pattern of intake compared to their respective control conditions. Although TBZ did not significantly change the total grams of pellets consumed, it did significantly increase time spent eating at the highest dose. This is important because previous behavioral studies indicate that time allocated to a particular activity is a fundamental marker of reinforcement value (Baum and Rachlin, 1969). Previous studies have shown that administration of TBZ had no significant effect on free food intake in both mice and rats (Nunes et al., 2013; Pardo et al., 2015; Correa et al., 2018; López-Cruz et al., 2018) and did not induce changes in hedonic taste reactivity to sucrose (Pardo et al., 2015). In rats responding on effort-based choice tasks involving lever pressing, TBZ-induced decreases in lever pressing are actually accompanied by increased intake of the concurrently available chow (Nunes et al., 2013; Yohn et al., 2016b). Taken together, these data are consistent with other studies indicating that the TBZ-induced relative shifts in choice behavior do not appear to be due to changes in primary food motivation (Randall et al., 2012, 2014; Nunes et al., 2013). Moreover, considerable evidence indicates that nucleus accumbens DA depletions do not impair the unconditioned reinforcing properties of food (Koob et al., 1978; Salamone et al., 1991, 2016; Salamone and Correa, 2002, 2012; Kelley et al., 2005). Thus, the T-maze choice paradigm appears to be useful for exploring changes in patterns of food consumption (rather than total food consumed), as well as changes in preferences after conditions that impair voluntary exercise.

We also tried to alter the value of the least preferred reinforcer, the fruit odor. There is considerable evidence showing an increase in sexual motivation of male rodents when a female odor is present (Portillo and Paredes, 2004; Trezza et al., 2011). In rodents, olfactory stimuli from conspecific estrous females can be conditional cues that induce incentive motivation and seeking behavior parallel to a significant increase in accumbens DA (Fujiwara and Chiba, 2018). Thus, we increased the value of the odor by using social odors (conspecific male and conspecific female). All the subjects were male, and the male odor significantly increased time sniffing the cotton ball but did not produce a significant reduction in time running. However, when the cotton contained a female odor, male mice increased sniffing time almost 10-fold, and this increase was paralleled by a significant reduction in time spent running, but not time eating. These results were parallel for the time-in-compartment variable. However, the presence of these social odors made animals more active in exploring the T-maze: mice increased entries in all the compartments when conspecific odors substituted the fruit odor. Thus, in the present results, male mice showed a decrease in the relative preference for the RW when a female odor was present, making food and opposite-sex odor equally preferred (around 30 s of interaction with each stimulus).

Finally, in order to reduce time in the RW, we tried to devalue the RW stimulus in different ways. Devaluing the RW by making it more resistant to turn and thus increasing muscular effort produced a very different pattern of effects compared to TBZ. Animals reduced time running, but there was no compensatory increase in time eating. In fact, animals tended to increase the time spent in the RW compartment, indicating no avoidance of the RW, and no increased interest in the other stimuli. Thus, force requirement does not seem to be the key factor underlying the effects of TBZ on RW preference in the present experiments. This result is similar to previous experiments using weights attached to a lever to increase resistance in an operant setting (Ishiwari et al., 2004). In that experiment, rats with a local nucleus accumbens DA depletion were relatively insensitive to different force requirements but were very sensitive to temporal or rate components of work requirements as compared to controls.

The term fatigue has been used for many different aspects of human performance. Fatigue includes a range of effects that can vary between reductions in physical and cognitive functions that extend from an exercise-induced impairment of motor performance to the sensations of tiredness and weakness that accompany some clinical conditions (Enoka and Duchateau, 2016). In order to assess all these components, most authors have established a dichotomy between central and peripheral fatigue or perceived fatigability and performance fatigability (Enoka and Stuart, 1992; Enoka and Duchateau, 2016). Among perceived fatigability, psychological factors, and in particular motivational factors, play a role in healthy individuals (McCormick et al., 2015), but they play an even more prominent role in the type of fatigue reported in various clinical conditions, such as in Parkinson’s disease (Kluger et al., 2013) and in depressed patients (Caligiuri and Ellwanger, 2000; Demyttenaere et al., 2005; Fava et al., 2014). Interestingly, this type of fatigue in Parkinson’s disease is significantly correlated with level of depression (Kluger et al., 2013). In summary, it seems that the type of fatigue generated by DA depletion, rather than a decline in muscle force, is more likely to reduce the capacity to sustain high levels of voluntary activation.

The FST is the classical animal test to evaluate antidepressant actions of different types of drugs (Armario et al., 1988; Lucki, 1997). This test, traditionally, provides information about passive behaviors such as immobility (Porsolt et al., 1977), as a measure of behavioral despair or “giving up,” but it also can provide information about behaviors directed to the maintenance of vigorous and persistent active responding in order to escape (Gil and Armario, 1998). In fact, this test provides information about different parameters of behavioral activation such as climbing and swimming (Armario et al., 1988; Gil and Armario, 1998; Slattery and Cryan, 2012). In the present setting, the administration of a broad range of TBZ doses that have previously been shown to reduce DA levels in the ventral striatum of mice (López-Cruz et al., 2018) dose-dependently produced a significant decrease in climbing (active behavior) and also increased time spent immobile in the FST (Figure 8). Previous studies have shown that after chronic oral administration of TBZ by putting it in the food, the immobility of mice in the FST increased significantly (Wang et al., 2010). Moreover, D2 receptor antagonists not only increased immobility in rats but also concurrently decreased active climbing (Gil and Armario, 1998; Li et al., 2015). Thus, DA depletion in the T-maze RW test (a test of preference for rewarding stimuli with different vigor requirements) and also in the FST (a test of non-escapable stress; Armario and Nadal, 2013) reduced behavioral activation.

In addition, because aversive states such as stress and anxiety could be factors that play a role in T-maze procedures and since it has been previously demonstrated that, in operant paradigms of effort-based choice in rats, acute stress decreased preference for the more costly reward (Shafiei et al., 2012), we performed a T-maze experiment in which we reduced the value of the RW by placing over it an intense light, like the one used in anxiety paradigms, thus making it more aversive. Indeed, animals spent less time running and tended to spend more time engaging with the other two stimuli, although only eating was statistically significant. However, the pattern of results for the other two variables was quite different from the results found with TBZ: animals significantly reduced time spent in the RW compartment and increased time spent in the other two compartments. They also increased entries into those other two compartments. Thus, animals not only reduced significantly the act of running but also spent less time in close proximity of the RW and explored the other two stimuli (not only the food) more. Moreover, TBZ did not produce any significant effect on anxiety-related parameters, such as time spent in the lit chamber of the DL box or time spent in the open arms of the EPM paradigm (Table 3). These results are in accordance with previous studies in which the administration of a high dose of TBZ (8.0 mg/kg) did not affect anxiety in the DL box (Correa et al., 2018) and administration of the D2 antagonist haloperidol did not produce any anxiogenic effect in mice assessed in the EPM paradigm (Pail et al., 2015). However, in the present studies, the administration of the two highest doses of TBZ (6.0 and 8.0 mg/kg) significantly decreased the total number of crosses between compartments in the DL box and total arm entries in the EPM paradigm. These are parameters that, while affected by the aversive nature of some areas in the paradigm, are considered to be more related to spontaneous locomotion.

In summary, the present work confirms that the three-choice T-maze task, in which rodents show a high preference for exercise, is sensitive to pharmacological and motivational manipulations and that mice allocate their behavior from one stimulus to another depending on a number of different conditions. TBZ administration induces changes in preferences based on effort requirements, and those effects do not closely resemble changes in preference produced by altering the force requirements or enhancing the reinforcing value of food. While making the RW more aversive by shining a light on it produced some effects that are similar to TBZ (e.g., reducing time spent running and increasing eating), these manipulations do not produce the same effects on other measures (e.g., time in RW compartment). TBZ also reduced climbing in the FST, indicating that TBZ generally reduces highly active behaviors. Numerous studies have reported that TBZ and DA antagonists affect effortful behaviors in rodents when animals have to work (lever pressing or climbing a barrier) in order to obtain a high-value reinforcer when they concurrently have a free low-value reinforcer (Pardo et al., 2012, 2015; Nunes et al., 2013; Randall et al., 2014; Yohn et al., 2015a,b, 2016a,b; Contreras-Mora et al., 2018; Correa et al., 2018; Rotolo et al., 2019; Yang et al., 2020). In conclusion, the three-choice T-maze task can evaluate preference for exercise and anergia induced by DA impaired function, and this task could have potential clinical relevance for modeling the psychomotor retardation, anergia, fatigue, and the low-effort bias seen in some human psychopathologies.

## Data Availability Statement

The raw data supporting the conclusions of this article will be made available by the authors, without undue reservation, to any qualified researcher.

## Ethics Statement

The animal study was reviewed and approved by Institutional Animal Care and Use committee of Universitat Jaume I.

## Author Contributions

CC-R: performed experiments, analysis of data and writing of manuscript. LL-C: performed experiments, and analysis of data. NS, PI-M and AM-V: performed different experiments. JS: design of experiments and writing of manuscript. MC: design of experiments, supervision of experimental phase, analysis of data and writing of manuscript.

## Conflict of Interest

The authors declare that the research was conducted in the absence of any commercial or financial relationships that could be construed as a potential conflict of interest.
